# Experiences with image quality and radiation dose of cone beam computed tomography (CBCT) and multidetector computed tomography (MDCT) in pediatric extremity trauma

**DOI:** 10.1007/s00256-020-03506-9

**Published:** 2020-06-14

**Authors:** Sebastian Tschauner, Robert Marterer, Eszter Nagy, Georg Singer, Michael Riccabona, Erich Sorantin

**Affiliations:** 1 Division of Pediatric Radiology, Department of Radiology, Medical University of Graz, Auenbruggerplatz 34, 8036 Graz, Austria; 2 Department of Paediatric and Adolescent Surgery, Medical University of Graz, Auenbruggerplatz 34, Graz, 8036 Austria

**Keywords:** Humans, Child, Multidetector computed tomography, Cone-beam computed tomography, Musculoskeletal diseases

## Abstract

**Introduction:**

Novel dedicated extremity cone beam computed tomography (CBCT) devices, recently introduced to the market, raised attention as a possible alternative in advanced diagnostic pediatric trauma imaging, today usually performed by multidetector computed tomography (MDCT). This work aimed to compare image quality and radiation dose of CBCT and MDCT.

**Materials and methods:**

Fifty-four CBCT-MDCT examination pairs, containing nine MDCTs acquired in parallel prospectively and 45 MDCTs matched in retrospect, were included in this study. Image quality was analyzed semi-objectively by measuring noise, contrast-to-noise ratio (CNR), and signal-to-noise ratios (SNR) and subjectively by performing image impression ratings. CT dose records were readout.

**Results:**

Image noise was significantly lower in CBCT compared with MDCT, both semi-objectively and subjectively (both *p* < 0.001). CNR and SNRs were also in favor of CBCT, though CBCT examinations exhibited significantly more beam hardening artifacts that diminished the advantages of the superior semi-objective image quality. These artifacts were believed to occur more often in children due to numerous bone-cartilage transitions in open growth plates and may have led to a better subjective diagnostic certainty rating (*p* = 0.001). Motion artifacts were infrequently, but exclusively observed in CBCT. CT dose index (CTDI_vol_) was substantially lower in CBCT (*p* < 0.001).

**Conclusion:**

Dedicated extremity CBCT could be an alternative low-dose modality in the diagnostic pathway of pediatric fractures. At lower doses compared with MDCT and commonly affected by beam hardening artifacts, semi-objective CBCT image quality parameters were generally better than in MDCT.

**Electronic supplementary material:**

The online version of this article (10.1007/s00256-020-03506-9) contains supplementary material, which is available to authorized users.

## Introduction

Cone beam computed tomography (CBCT) represents a well-established, widely available, and often used modality in dental imaging for many years [1, 2]. Recently, some manufacturers introduced dedicated extremity CBCT scanners [3–6]. Their novelty involves an element of uncertainty concerning the advantages and drawbacks of these new devices, underlining the necessity to obtain more related scientific data in comparison with commonly performed multidetector computed tomography (MDCT).

The advertised benefits of CBCT scanners usually contain compact constructions, device mobility, and expected dose reduction with preserved image quality [5–8]. Hence, pediatric extremity CBCT offers possible dose savings that are of concern due to a higher radiation sensitivity of children [9].

Initial studies and experiences with extremity CBCT machines in adults and children consistently demonstrated excellent image quality and diagnostic accuracy at comparable levels to commonly used MDCT scanners [3, 4, 10–12]. Moreover, reported doses were significantly lower in CBCT compared with MDCT [3–5, 8, 13, 14]. Authors characterized CBCT as a valuable method in suspected scaphoid fractures, even though it was not able to find or exclude a fracture in every case [15]. However, MDCT-related literature provided similar results of occult fractures that only became evident on further or follow-up imaging [16].

The current study compared semi-objective and subjective image quality parameters and dose records in pairs of CBCT and MDCT extremity examinations of acutely injured pediatric patients.

## Materials and methods

**Fig. 1:**
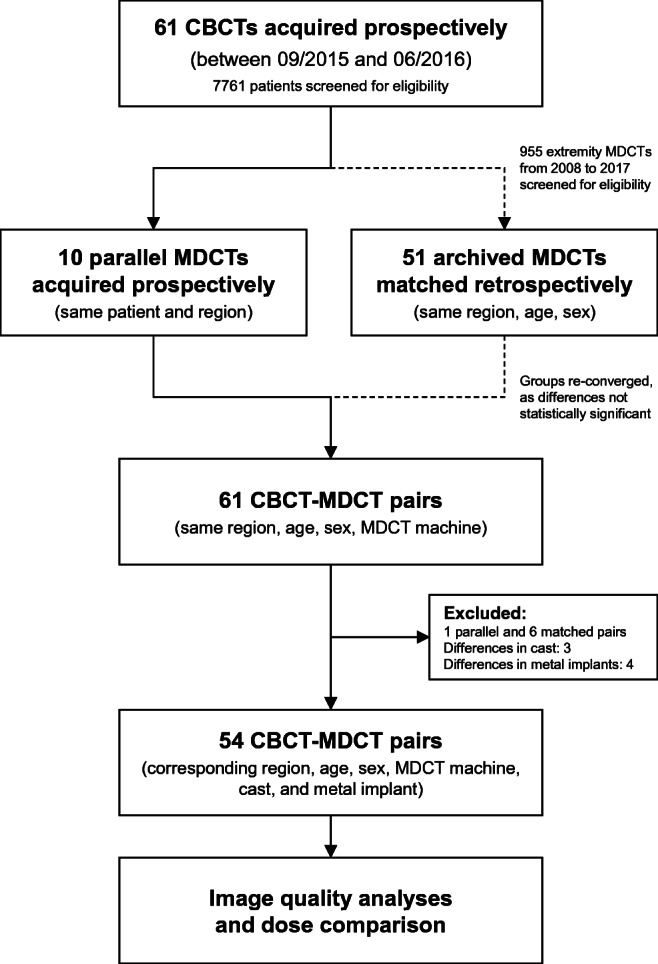
Flowchart of study-related events. All MDCTs had been performed on the same device. In the case of non-parallel acquisitions, the examinations were manually matched to the CBCTs based on region, age, sex, and the presence of casts or metal implants

Fifty-nine injured children were referred to a total of 61 CBCT examinations between September 2015 and June 2016, subsequently performed at the local division of pediatric radiology. In consenting patients, radiological technologists performed these CBCTs instead of the usually conducted MDCTs. A subsample of ten patients agreed to undergo an MDCT examination in parallel following written informed consent. We retrospectively matched the remaining 51 CBCTs to archived MDCTs of the same extremity region, age, and sex, all performed with the same study MDCT device. Seven of the 61 CBCT-MDCT pairs were excluded due to discrepancies in the presence of casts or metal implants, or the unavailabilty of a proper study for matching. Fifty-four study pairs remained for further comparisons (wrist *n* = 19, ankle *n* = 11, elbow *n* = 9, finger *n* = 6, foot *n* = 5, hand *n* = 3, knee *n* = 1). Figure 1 details the above-described recruitment process leading to a mean patient age of 14.3 ± 2.2 years in CBCT vs. 14.4 ± 2.2 years in MDCT, each group containing 24 females and 30 males. Age differences did not reach statistical significance (*p* = 0.832).

Technologists acquired the CBCTs with a Planmed Verity scanner (Planmed Oy, Helsinki, Finland) by centering the examined body part in the middle of the gantry, as indicated by the integrated laser position markers. The acquired cylindrical field of view (FOV) extended 12 or 6 cm proximo-distally, with a diameter of 16 cm. The preset unchangeable acquisition time was 36 s in a 210° rotation. Image reconstructions contained three sectional planes relative to the axis of the examined body part (axial, coronal, sagittal). As recommended, the device-fitted lead curtain shield was used [17] in addition to the routinely applied body shielding whenever possible. The corresponding MDCT was an Aquilion One (Toshiba Medical Systems Corporation, Otawara-shi, Japan). The injured extremity was centered in the scanner gantry as effectively as possible, before every volumetric image acquisition without table increment. Technologists performed scout images before MDCT scanning. Proximo-distal scan field extension varied from 10 to 16 cm with variable diameters. Tube rotation time was 0.5 s. Table 1 lists the acquisition and reconstruction settings.

**Table 1:** Acquisition and image reconstruction settings used in CBCT and MDCT

		CBCT (Planmed Verity)	MDCT (Toshiba Aquilion One)	Significance (*p*)
Image acquisition	Field of view (mm)	160 × 160	120 × 120	–
	CTDI_vol_ (16 cm phantom) mGy (mean SD)	2.3 ± 0.8	3.2 ± 1.0 without AIDR3D 4.1 ± 1.0 with AIDR3D 2.9 ± 0.7	*p* < 0.001 *p < 0.001* *p < 0.001*
	DLP mGy*cm (mean ± SD)	27.9 ± 11.9	34.8 ± 18.1 without AIDR3D 45.4 ± 21.9 with AIDR3D 30.3 ± 14.3	*p* = 0.021 *p < 0.001* *p = 0.378*
	kVp (mean ± SD)	91.0 ± 3.5	120.0 ± 2.7	*p* < 0.001
	mA (mean ± SD)	4.6 ± 1.4	37.6 ± 11.3	*p* < 0.001
	mAs (mean ± SD)	27.6 ± 8.5	19.0 ± 5.8	*p* < 0.001
	Rotation/exposure time (seconds)	6.0/36.0	0.5/0.5	*p* < 0.001
	Slice thickness (mean ± SD)	1.3 ± 0.2 mm	1.3 ± 0.3 mm	*p* = 0.104
Image reconstruction	Planes	Axial, coronal, sagittal	Axial, coronal, sagittal	–
	Kernel	Sharp	FC 18 (AIDR3D (after 2012))/STD/W/CB	–
	Pixel matrix	800 × 800	512 × 512	–
	Pixel spacing (mean ± SD)	0.2 ± 0.0 mm	0.2 ± 0.1 mm	–

The primary goal of the current analyses was to compare both devices in a realistic pediatric trauma setting. Our department’s standard MDCT imaging protocols used in this study had been long-time optimized to compound for acceptable image quality at low dose. To facilitate comparisons on equal terms, we adapted and lowered the pre-saved exposure protocols of the CBCT device based on phantom and cadaveric experiments [13] before the initiation of the patient recruitment phase. Dataset 1 contains detailed exposure settings of all included examinations.

**Fig. 2:**
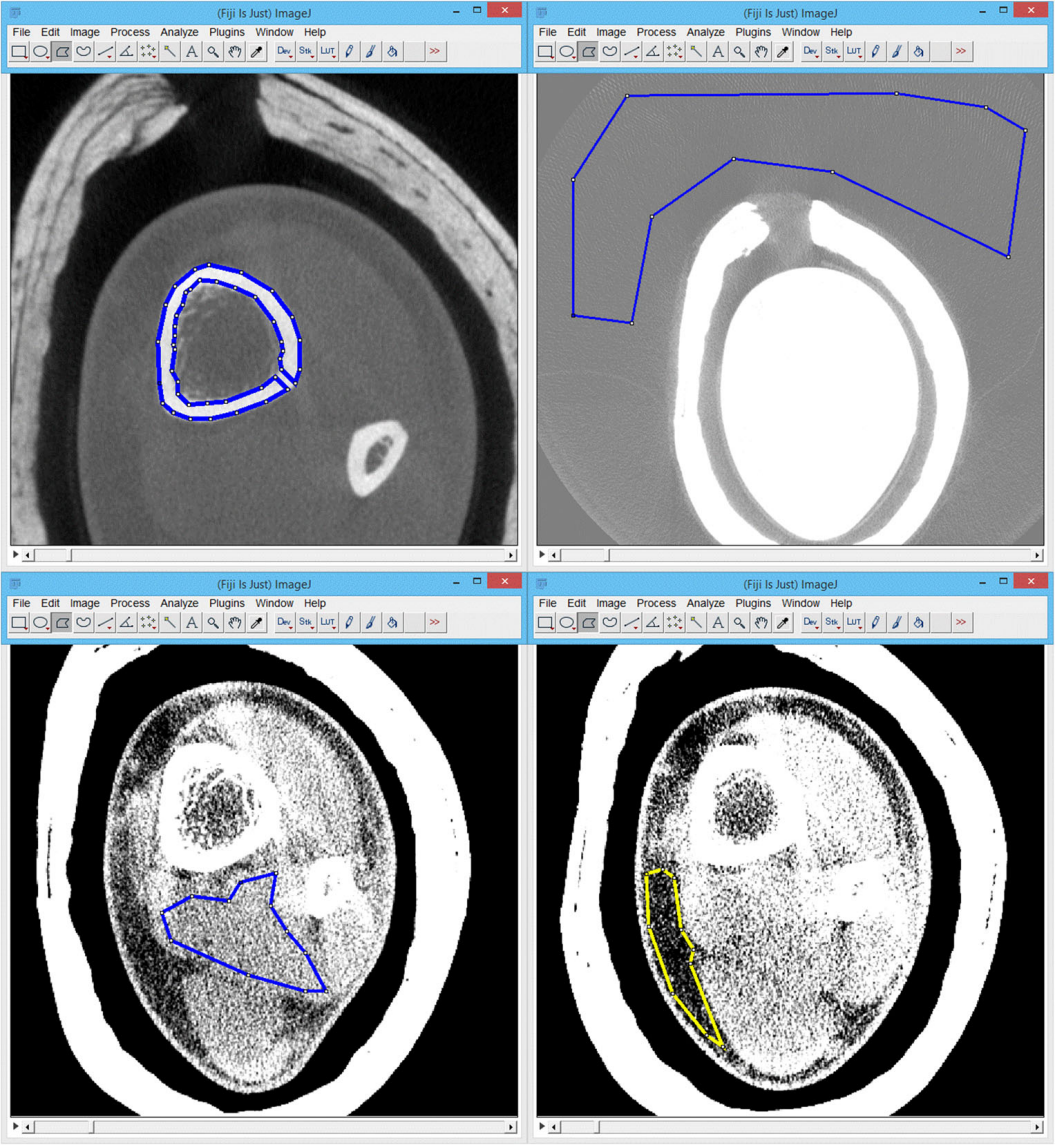
The procedure of ROI measurements utilizing a polygonal selection tool in FIJI shown for cortical bone (top left), air (top right), muscle (lower left), and fat (lower right) in a sample CBCT of an ankle

Accurate quantitative image quality comparisons between the two different modalities are nearly impossible to perform, suffering from fluctuating non-calibrated CBCT grayscale values [18–20], their kVp dependencies [20–23], and principal scanner differences [21, 24, 25]. Matching pairs of kVp settings are not available on the examined machines. Therefore, we decided to assess semi-objective and subjective image quality parameters, the former by opening and arranging the corresponding CBCT and MDCT examinations side by side in Fiji 1.49v (a distribution of the open source image processing software ImageJ, http://rsbweb.nih.gov/ij/) [26]. The first author (S.T., radiology resident with 6 years of experience) manually placed polygonal regions of interest (ROI) in corresponding axial image slices to retrieve the mean and standard deviation of three repeated measurements of cortical bone, fat, muscle, and air, as displayed in Fig. 2. Moreover, we read out histograms of every axial image stack to get the peaks of air, soft tissue, cortical bone, and the maximum pixel intensity. The cortical bone peak of the parallel CBCT-MDCT examination subsample served as a correction reference to compensate for the aforementioned methodological differences in grayscale value display [20, 23, 27], marked with the term “HU_corr_” in CBCT throughout the manuscript. The corrected standard deviation of the image background (air) acted as metric for image noise. Contrast-to-noise ratio (CNR = [MEAN cortical bone−MEAN air]/SD air) and signal-to-noise ratios (SNR = MEAN tissue/SD tissue) of cortical bone, fat, and muscle functioned as additional semi-objective image quality parameters.Three observers (S.T., R.M., and E.N. with respective extremity CT experiences of 6, 8, and 4 years) separately rated subjective image impression on a five-grade Likert scale (1 = very good, 2 = good, 3 = fair, 4 = poor, 5 = very poor) in a dark reading room, anonymized, randomly sorted, and in reader-desired grayscale window settings. Color-calibrated 4-megapixel RadiForce RX440 monitors (Eizo, Hakusan, Japan) displayed the images opened with the local picture archiving and communication system (PACS) software syngo.plaza version VB20A (Siemens Healthineers, Erlangen, Germany). The observers entered their image impression ratings including diagnostic certainty, image details, sharpness, and contrast, as well as artifacts in a form as shown in Table 2.

**Table 2:** Subjective image interpretation. Questions were answered by the observers anonymized and randomized. Differences between CBCT and MDCT and interrater correlation coefficients are given within the modalities. *Mean and SD instead of median and range

				Group differences (median tests) between CBCT and MDCT					Interrater differences (ICC(3,k)) separate for CBCT and MDCT			
Category	Parameter	Possible answers	Modality	Median (mean*) (parallel/matched)	Range (SD*) (parallel/matched)	Min (parallel/matched)	Max (parallel/matched)	Significance (*p*)	ICC	95% CI (lower limit)	95% CI (upper limit)	Significance (*p*)
General	Fracture	Yes or no	CBCT	0.65*	0.48*	0	1	*p* = 0.176	0.83	0.74	0.90	*p* < 0.001
			MDCT	0.76*	0.39*	0	1		0.88	0.80	0.92	*p* < 0.001
	Diagnostic certainty	(Very good) 1–5 (very poor)	CBCT	1	4	1	5	*p* < 0.001	0.73	0.58	0.84	*p* < 0.001
			MDCT	1 (1/1)	1 (0/1)	1 (1/1)	2 (1/2)		0.40	0.06	0.63	*p* = 0.012
Image quality	Overall image quality	(Very good) 1–5 (very poor)	CBCT	3	3	2	5	*p* < 0.001	0.67	0.29	0.83	*p* < 0.001
			MDCT	2 (2/2)	3 (2/3)	1 (1/1)	4 (3/4)		0.66	0.42	0.81	*p* < 0.001
	Sharpness	(Very good) 1–5 (very poor)	CBCT	2	2	1	3	*p* = 0.013	0.43	0.10	0.65	*p* < 0.001
			MDCT	2 (2/2)	2 (2/2)	1 (1/1)	3 (3/3)		0.39	− 0.02	0.65	*p* < 0.001
	Contrast	(Very good) 1–5 (very poor)	CBCT	2	1	1	2	*p* < 0.001	0.17	− 0.09	0.41	*p* = 0.063
			MDCT	1 (1/1)	1 (1/2)	1 (1/1)	3 (2/3)		0.63	0.35	0.78	*p* < 0.001
	Detail resolution	(Very good) 1–5 (very poor)	CBCT	1	2	1	3	*p* = 0.005	0.20	− 0.19	0.48	*p* = 0.138
			MDCT	2 (2/2)	2 (2/2)	1 (1/1)	3 (3/3)		0.36	0.04	0.59	*p* = 0.014
	Bone (cortical)	(Very good) 1–5 (very poor)	CBCT	1	2	1	3	*p* = 0.316	0.08	− 0.13	0.30	*p* = 0.209
			MDCT	1 (1/1)	1 (0/1)	1 (1/1)	2 (1/2)		0.12	− 0.14	0.36	*p* = 0.164
	Bone (trabecular)	(Very good) 1–5 (very poor)	CBCT	2	2	1	3	*p* < 0.001	0.39	0.07	0.62	*p* = 0.001
			MDCT	2 (2/2)	3 (2/3)	1 (1/1)	4 (3/4)		0.53	0.28	0.71	*p* < 0.001
	Joints	(Very good) 1–5 (very poor)	CBCT	1	3	1	4	*p* = 0.143	0.09	− 0.08	0.28	*p* = 0.096
			MDCT	1 (1/1)	1 (1/1)	1 (1/1)	2 (2/2)		0.08	− 0.10	0.27	*p* = 0.168
	Soft tissue	(Very good) 1–5 (very poor)	CBCT	3	3	2	5	*p* = 0.187	0.24	− 0.07	0.50	*p* < 0.001
			MDCT	3 (3/3)	2 (2/2)	2 (2/2)	4 (4/4)		0.23	− 0.07	0.49	*p* < 0.001
Artifacts	Aliasing	(None) 1–5 (severe)	CBCT	1	1	1	2	*p* < 0.001	0.01	− 0.20	0.24	*p* = 0.452
			MDCT	2 (2/2)	4 (2/4)	1 (1/1)	5 (3/5)		0.56	0.07	0.78	*p* < 0.001
	Beam hardening (including streak, cupping, and dark band artifacts)	(None) 1–5 (severe)	CBCT	3	3	2	5	*p* < 0.001	0.51	0.06	0.74	*p* < 0.001
			MDCT	1 (1/1)	0 (0/0)	1 (1/1)	1 (1/1)		− 0.08	− 0.89	0.38	*p* = 0.610
	Motion	(None) 1–5 (severe)	CBCT	1	4	1	5	*p* = 0.013	0.90	0.84	0.94	*p* < 0.001
			MDCT	1 (1/1)	0 (0/0)	1 (1/1)	1 (1/1)		–	–	–	–
	Noise	(None) 1–5 (severe)	CBCT	2	2	1	3	*p* < 0.001	0.29	− 0.04	0.54	*p* = 0.035
			MDCT	3 (3/3)	3 (2/3)	2 (2/2)	5 (4/5)		0.54	0.29	0.72	*p* < 0.001
	Ring	(None) 1–5 (severe)	CBCT	1	0	1	1	*p* = 0.495	− 0.02	− 0.78	0.41	*p* = 0.533
			MDCT	1 (1/1)	2 (1/2)	1 (1/1)	3 (2/3)		0.88	0.82	0.93	*p* < 0.001

The authors analyzed the collected data in SPSS Statistics Version 21 (IBM Corp., Armonk, NY, USA) using descriptive statistics, as well as *t* test mean value comparisons in cases of ascertained, and nonparametric Mann–Whitney *U* tests in cases of lacking normal distributions. We calculated inter-observer agreement with ICC (3,k) (absolute agreement, two-way mixed average measures) for scales. *p* values lower than 0.050 were regarded to be statistically significant.

The local ethical review committee of the Medical University of Graz (IRB00002556) approved the study (No. EK 27-452 ex 14/15) and required written informed patient and parent consent before every study-related CBCT examination.

## Results

Nonparametric testing showed no significant differences between parallel and matched MDCTs regarding corrected noise (*p* = 0.250), CNR (*p* = 0.880), SNR (bone *p* = 0.825, fat *p* = 0.250, muscle *p* = 0.280), and dose (CTDI_vol_ *p* = 0.269, DLP *p* = 0.478). We, therefore, decided to reconverge all MDCTs and analyzed them as a single group.

**Fig. 3:**
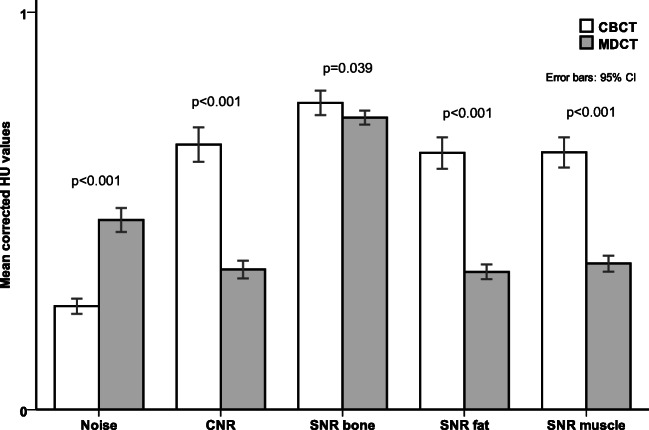
Bar chart displaying a set of different semi-objective image quality parameters. Mean corrected noise, contrast-to-noise ratio (CNR), and signal-to-noise ratios (SNR) in CBCT and MDCT. Error bars display 95% confidence intervals (CI). *Y* scale normalized from 0 to 1. Lower bars are better. *p* values are given for the respective examination pairs

Mean corrected background image noise was significantly lower in CBCT than in MDCT examinations (28.4 HU_corr_ vs. 52.2 HU, *p* < 0.001). CNR showed significant differences in favor of CBCT (*p* < 0.001). In CBCT, mean normalized CNR was 112.1 ± 26.6 HU_corr_ and in MDCT 59.3 ± 13.5 HU (*p* < 0.001). Significantly different normalized SNRs were found for cortical bone (CBCT = 2053.8 ± 303.3 HU_corr_ vs. MDCT = 1954.6 ± 172.8 HU, *p* = 0.039), fat (CBCT = 38.8 ± 8.4 HU_corr_ vs. MDCT = 21.3 ± 4.0 HU, *p* < 0.001), and muscle (CBCT = 37.9 ± 8.2 HU_corr_ vs. MDCT = 21.5 ± 4.3 HU, *p* < 0.001). Figure 3 graphically depicts the parameters mentioned above.

**Fig. 4:**
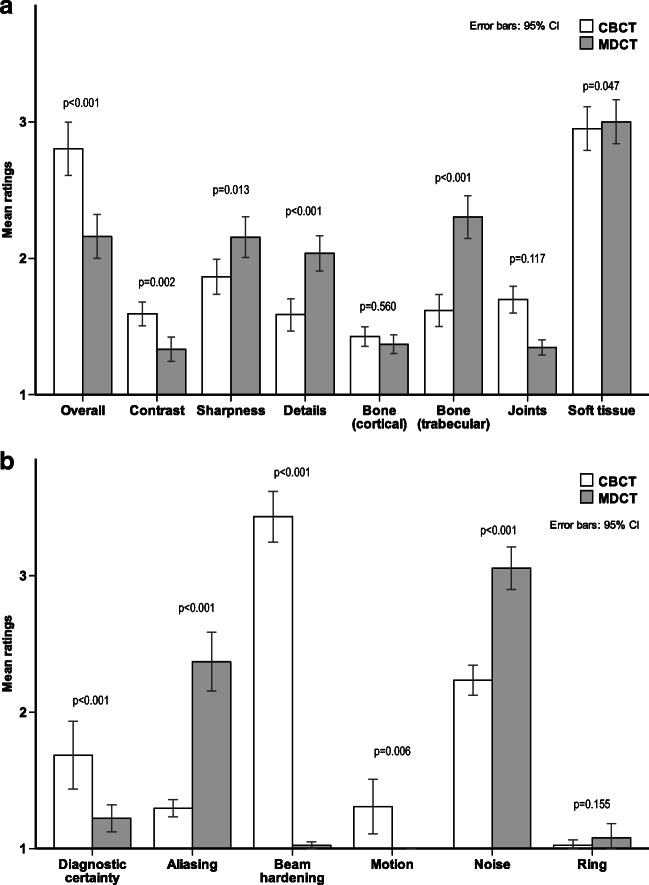
Subjective image impression ratings. **a** Mean image quality and artifacts, independently rated by three pediatric radiologists. **b** Diagnostic certainty ratings and image artifacts. Lower bars are better. *p* values are given for the respective examination pairs

**Fig. 5:**
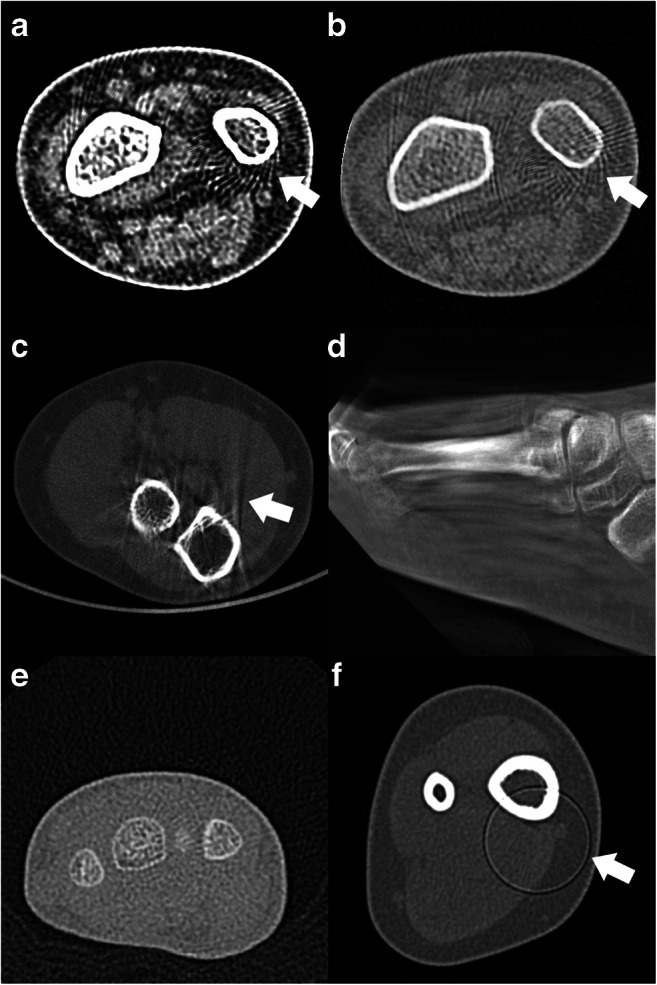
Examples of various CT artifacts. **a** and **b** Aliasing artifacts in an MDCT of the wrist. **c** Beam hardening artifacts in an elbow CBCT. **d** Motion artifacts in a CBCT of the foot. **e** Image noise in a wrist MDCT. **f** Ring artifact in an ankle MDCT

Median subjective overall image quality did differ significantly (CBCT = 3, range 2–5 vs. MDCT = 2, range 1–4 points, *p* < 0.001), as did diagnostic certainty (CBCT = 1, range 1–5 vs. MDCT = 1, range 1–2 points, *p* < 0.001). Sharpness, details, and depiction of trabecular bone were significantly better rated in MDCT, contrast, and joints in CBCT. The observers scored cortical bone and soft tissue without significant differences in the assessed bone kernel. Figure 4 displays all image impression scorings.Beam hardening artifacts were consensually rated more severe in CBCT. In median, these artifacts were valued at 3 (range 2–5 points) in CBCT and 1 (range 1–1 point) in MDCT (*p* < 0.001). On the other hand, image noise was superior in CBCT images with a median rating of 2 (range 1–3) vs. 3 (range 2–5) points (*p* < 0.001) and the raters recorded less aliasing (1, range 1–2 vs. 2, range 1–5 points, *p* < 0.001). Ring artifacts were rarely seen and did not differ significantly between the devices. Motion artifacts only appeared in CBCT (1, range 1–5 points; and 1, range 1–1 point, *p* = 0.013). There was a significant linear correlation between both subjective and semi-objective image noise ratings (*R* = 0.631, *p* < 0.001), between pixel spacing and sharpness impression (*R* = -0.231, *p* = 0.016), and between CNR and contrast perception (*R* = 0.222, *p* = 0.021). The mentioned artifacts are exemplarily shown in Fig. 5.

Interrater correlation indicated variable agreements, explicitly listed in Table 2. We noted satisfying observer accordance in fracture detection, diagnostic certainty, overall image quality, and motion and ring artifacts. The remaining variables showed ambivalent amounts of consensus.

Mean CTDI_vol_ (16 cm phantom) was 2.3 ± 0.8 mGy in CBCT and 3.2 ± 1.0 mGy in MDCT (*p* < 0.001), while DLP was 27.9 ± 11.9 mGy*cm in CBCT and 34.8 ± 18.1 mGy*cm in MDCT (*p* = 0.021), each significantly lower in CBCT. The study MDCT machine was updated with iterative reconstruction algorithms (AIDR3D) in 2012, resulting in a significant CTDI_vol_ and DLP drop from mean 4.1 ± 1.0 mGy to 2.9 ± 0.7 mGy (*p* < 0.001) and mean 45.4 ± 21.9 mGy*cm to 30.3 ± 14.3 mGy*cm (*p* = 0.004) at virtually constant image quality values. When excluding the 16 MDCT examinations without iterative reconstruction, CTDI_vol_ values still were significantly lower (*p* < 0.001) in CBCT than in MDCT, whereas DLPs did no longer differ significantly (*p* = 0.378). For further information, please refer to Table 1 and Dataset 1.

## Discussion

This study compared the image quality of an extremity CBCT and an MDCT in a small pediatric trauma patient sample. CBCT achieved significantly lower radiation doses at superior semi-objective image quality, whereas beam hardening and to a certain extent also motion artifacts affected the subjective image impression.

In children, the single directly related study by Pugmire et al. retrospectively investigated a CBCT device in the setting of pediatric foot and ankle injuries. The authors found that CBCT was able to detect clinically relevant information and was, compared with MDCT, considered a low-dose alternative [3]. Huang et al. [4] and Demehri et al. [11] published comparable findings in adults before. The limited extent of available literature demonstrates the importance of further research in the field of pediatric extremity CBCT.

Koivisto et al. previously reported exceptionally low radiation doses of the dedicated extremity CBCT machine used in the current study [5, 8]. Given these promising results, we decided to scan a consenting patient subsample parallelly on both devices with optimized exposure settings. Effective radiation doses in extremity CT are known to decrease further away from the torso [28], predestining cross-sectional imaging modalities like CBCT and MDCT for body regions like the hand or foot, where superpositions in radiography commonly compromise diagnostic accuracy [29]. In this specific paper, we did not conduct surface dose measurements or estimations of effective doses, as too many unknown factors influence reliable risk approximations [30]. These factors include a variable distribution of radio-sensitive red and relatively radio-insensitive yellow bone marrow, which are known to decrease until adulthood [31] and are prone to inter-individual differences [32]. Moreover, the concept of effective dose is not a valid measure of individual patient risk [33], stochastic radiation damage may be underpredicted [34, 35], and suitable pediatric tissue weighting factors remain a topic of ongoing discussion [36, 37]. Given the mentioned drawbacks, we decided to report CTDI_vol_ and DLP values only, which both were significantly in favor of CBCT.

General image impression differed between the modalities, and a trained eye recognized the underlying diagnostic device with ease, primarily based on noise characteristics and the presence of beam hardening artifacts. The latter was the most significant drawback of the CBCT images, while semi-objective image quality parameters were in benefit of CBCT. Open growth plates in children caused additional bone-cartilage transitions, which pronounced these beam hardening artifacts even more. New developments in the field of CBCT image processing using statistical iterative reconstruction algorithms [38, 39] may help to suppress beam hardening artifacts in the future. Maybe pediatric radiologists will also need to adapt to the unique visual appearance and the typical artifacts of CBCT, as it was necessary for dose-reduced noisy images and iterative dose-reconstruction in the last decades.

**Fig. 6:**
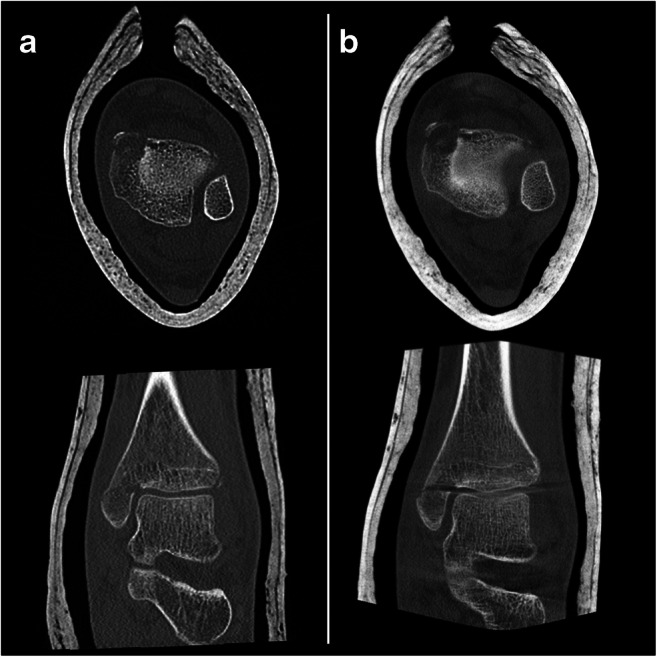
A side-by-side comparison of an MDCT examination of the right ankle in a 15-year-old girl with a distal tibial fracture. **a** Axial (top) and coronal (bottom) MDCT images. **b** Axial (top) and coronal (bottom) reconstructions of the corresponding CBCT examination. Especially the coronal slices show beam hardening artifacts in the image plane at bone-to-cartilage transitions

**Fig. 7:**
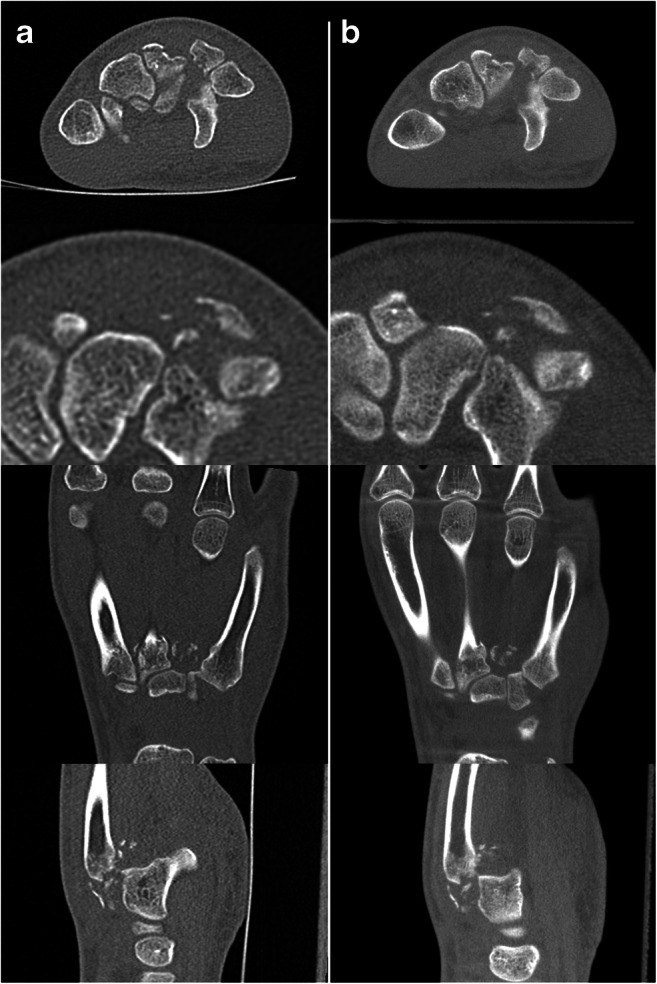
Example of a wrist CBCT (right column) and MDCT (left column) in a 17-year-old adolescent with comminuted fractures of the metacarpal bases and the distal row of carpals. First row axial, second row axial detail, third row coronal, fourth row sagittal reformations, all in 1-mm slice thickness

In the current manuscript, we did not assess diagnostic accuracy in greater detail due to the heterogeneity of the recruited patients and injuries and the fact that sufficient clinical and radiological follow-up was commonly not available in the partly retrospective study design. During follow-up, MRI revealed a single undisplaced scaphoid fracture, occult in a non-parallel wrist CBCT. However, due to the insufficient data situation, we decided not to give diagnostic accuracy values. Raters were significantly more confident to detect a fracture in MDCT examinations. Previous reports in the literature did not find significant diagnostic differences between the modalities [4, 10]. In the study by Faccioli et al. on the diagnostic accuracy of finger fractures in CBCT and MDCT, there was a lower count of detected bone fragments in CBCT, but these differences were not statistically significant [10]. Accordingly, our data indicated that the allocation of a particular voxel to its correct spatial position was harder in CBCT (compare Figs. 6 and 7), as a result of beam hardening artifacts. Other studies assessed the accuracy of CBCT, MDCT, and MRI, and reported missed fractures in all three modalities [15, 40].

The most critical weakness of the present study is caused by systematic differences between the machines, which prevent a direct objective comparison between CBCT and MDCT. So we tried to mitigate potential resulting biases, as side-by-side comparisons are needed. The grayscale values provided by CBCT are variable and cannot be correlated to MDCT HU values reliably, even when corrected. Technical conditions and exposure options differ substantially, and the chosen protocols could be considered inappropriate. Nevertheless, the modalities need to be evaluated in a clinical setting to reveal strengths, weaknesses, and areas of possible improvements. For example, it would have been impossible to analyze motion artifacts in phantoms or cadavers. Another limitation is the small sample size of parallel patients. We decided to scan only a limited consenting patient subsample, in order to minimize the radiation dose and to gain data to properly match the remaining CBCT studies. Due to systematic optimizations, the study-related additional dose equaled about 5 days of natural background radiation per patient, which, we believe, is justifiable in proportion to the gain of knowledge. Another limitation is the fact that imaging protocols were not identical, which is a general problem as both methods differ substantially regarding technical conditions and exposure options. It was not tried to achieve similar CTDI_vol_ or DLP values for both modalities forcefully, as previous studies indicated the low-dose capabilities of the used CBCT device [5, 8]. Also, it was not possible to apply identical reconstruction settings and parameters apart from slice thickness. Even though blinded observers rated subjective image quality, image impressions were unmistakably different, revealing the underlying imaging device to a qualified radiologist. We did not record examination durations and patient comfort in this study, but subjective impression and experience did not indicate clinically relevant time differences in either modality in this specific pediatric setting. This topic warrants future research, for instance, assessing patient discomfort and pain between CBCT and MDCT machines in extremity trauma examinations.

## Conclusion

In conclusion, dedicated trauma extremity CBCT may require lower radiation doses than MDCT at increased semi-objective image quality parameters. However, beam hardening artifacts might degrade the subjective image impression in many cases.

## Electronic supplementary material

ESM 1 Dataset 1 (XLSX 113 kb)

## Abbreviations

AIDR3D: Adaptive iterative dose reduction using three dimensional processing
CBCT: Cone beam computed tomography
CI: Confidence interval
DLP: Dose length product
mGy: Milligray
CNR: Contrast-to-noise ratio
CTDI_vol_: Computed tomography dose index volume
PACS: Picture archiving and communication system
SD: Standard deviation
SNR: Signal-to-noise ratio
